# Social media and mental health in students: a cross-sectional study during the Covid-19 pandemic

**DOI:** 10.1186/s12888-023-04859-w

**Published:** 2023-06-22

**Authors:** Abouzar Nazari, Maede Hosseinnia, Samaneh Torkian, Gholamreza Garmaroudi

**Affiliations:** 1 Department of Health Education and Promotion, Faculty of Health, Tehran University of Medical Sciences, Tehran, 1417613151 Iran; 2 Department of Health Education and Promotion, Faculty of Health, Isfahan University of Medical Sciences, Isfahan, Iran; 3 Department of Epidemiology and Biostatistics, Faculty of Health, Iran University of Medical Sciences, Tehran, 1417613151 Iran; 4 Department of Health Education and Promotion, School of Health, Tehran University of Medical Sciences, Tehran, 1417613151 Iran

**Keywords:** Social media, Mental Health, Students

## Abstract

**Background:**

Social media causes increased use and problems due to their attractions. Hence, it can affect mental health, especially in students. The present study was conducted with the aim of determining the relationship between the use of social media and the mental health of students.

**Materials and methods:**

The current cross-sectional study was conducted in 2021 on 781 university students in Lorestan province, who were selected by the Convenience Sampling method. The data was collected using a questionnaire on demographic characteristics, social media, problematic use of social media, and mental health (DASS-21). Data were analyzed in SPSS-26 software.

**Results:**

Shows that marital status, major, and household income are significantly associated with lower DASS21 scores (a lower DASS21 score means better mental health status). Also, problematic use of social media (β = 3.54, 95% CI: (3.23, 3.85)) was significantly associated with higher mental health scores (a higher DASS21 score means worse mental health status). Income and social media use (β = 1.02, 95% CI: 0.78, 1.25) were significantly associated with higher DASS21 scores (a higher DASS21 score means worse mental health status). Major was significantly associated with lower DASS21 scores (a lower DASS21 score means better mental health status).

**Conclusion:**

This study indicated that social media had a direct relationship with mental health. Despite the large amount of evidence suggesting that social media harms mental health, more research is still necessary to determine the cause and how social media can be used without harmful effects.

## Background

### Social media

Social media is one of the newest and most popular internet services, which has caused significant progress in the social systems of different countries in recent years [1, 2]. The use of the Internet has become popular among people in such a way that its use has become inevitable and has made life difficult for those who use it excessively [3]. Social media has attracted the attention of millions of users around the world owing to the possibility of fast communication, access to a large amount of information, and its widespread dissemination [4]. Facebook, WhatsApp, Instagram, and Twitter are the most popular media that have attractive and diverse spaces for online communication among users, especially the young generation [5, 6].

According to studies, at least 55% of the world’s population used social media in 2022 [7]. Iranian statistics also indicate that 78.5% of people use at least one social media. WhatsApp, with 71.1% of users, Instagram, with 49.4%, and Telegram, with 31.6% are the most popular social media among Iranians [8, 9].

The use of social media has increased significantly in all age groups due to the origin of the COVID-19 pandemic [10] .It affected younger people, especially students, due to educational and other purposes [11, 12]. Because of the sudden onset of the COVID-19 pandemic, educational institutions and learners had to accept e-learning as the only sustainable education option [13]. The rapid migration to E-learning has brought several challenges that can have both positive and negative consequences [14].

Unlike traditional media, where users are passive, social media enables people to create and share content; hence, they have become popular tools for social interaction [15].The freedom to choose to participate in the company of friends, anonymity, moderation, encouragement, the free exchange of feelings, and network interactions without physical presence and the constraints of the real world are some of the most significant factors that influence users’ continued activity in social media [16]. In social media, people can interact, maintain relationships, make new friends, and find out more about the people they know offline [17]. However, this popularity has resulted in significant lifestyle changes, as well as intentional or unintentional changes in various aspects of human social life [18]. Despite many advantages, the high use of social media brings negative physical, psychological, and social problems and consequences [19], but despite the use and access of more people to the Internet, its consequences and crises have been ignored [20].

### Use of social media and mental health

Spending too much time on social media can easily become problematic [21]. Excessive use of social media, called problematic use, has symptoms similar to addiction [22, 23]. Problematic use of social media represents a non-drug-related disorder in which harmful effects emerge due to preoccupation and compulsion to over-participate in social media platforms despite its highly negative consequences [24–26], which leads to adverse consequences of mental health, including anxiety, depression, lower well-being, and lower self-esteem [27–29].

### Mental health & use of social media

Mental health is the main pillar of healthy human societies, which plays a vital role in ensuring the dynamism and efficiency of any society in such a way that other parts of health cannot be achieved without mental health [30]. According to World Health Organization’s (WHO) definition, mental health refers to a person’s ability to communicate with others [31]. Some researchers believe that social relationships can significantly affect mental health and improve quality of life by creating a sense of belonging and social identity [32]. It is also reported that people with higher social interactions have higher physical and mental health [33].

Scientific evidence also shows that social media affect people’s mental health [34]. Social studies and critiques often emphasize the investigation of the negative effects of Internet use [35]. For example, Kim et al. studied 1573 participants aged 18–64 years and reported that Internet addiction and social media use were associated with higher levels of depression and suicidal thoughts [36]. Zadar also studied adults and reported that excessive use of social media and the Internet was correlated with stress, sleep disturbances, and personality disorders [37]. Richards et al. reported the negative effects of the Internet and social media on the health and quality of life of adolescents [38]. There have been numerous studies that examine Internet addiction and its associated problems in young people [39, 40], as well as reports of the effects of social media use on young people’s mental health [41, 42].

A study on Iranian students showed that social media leads to depression, anxiety, and mental health decline [25]. A study on Iranian students showed that social media leads to depression, anxiety, and mental health decline [25]. But no study has investigated the effects of social media on the mental health of students from a more traditional province with lower individualism and higher levels of social support (where they were thought to have lower social media use and better mental health) during the COVID-19 pandemic. As social media became more and more vital to university students’ social lives during the lockdowns, students were likely at increased risk of social media addiction, which could harm their mental health. University students depended more on social media due to the limitations of face-to-face interactions. In addition, previous studies were conducted exclusively on students in specific fields. However, in our study, all fields, including medical and non-medical science fields were investigated.

The present study was conducted to determine the relationship between the use of social media and mental health in students in Lorestan Province during the COVID-19 pandemic.

## Materials and methods

### Study design and participants

The current study was descriptive-analytical, cross-sectional, and conducted from February to March 2022 with a statistical population made up of students in all academic grades at universities in Lorestan Province (19 scientific and academic centers, including centers under the supervision of the Ministry of Health and the Ministry of Science).

### Sample size

According to the convenience sampling method, 781 people were chosen as participants in the present study. During the sampling, a questionnaire was created and uploaded virtually on Porsline’s website, and then the questionnaire link was shared in educational and academic groups on social media for students to complete the questionnaire under inclusion criteria (being a student at the University of Lorestan and consenting to participate in the study).

### Study tool

The research tools included the demographic information questionnaire, the standard social media use questionnaire, and the mental health questionnaire.

### Demographic information

The demographic information age, gender, ethnicity, province of residence, urban or rural, place of residence, semester, and the field of study, marital status, household income, education level, and employment status were recorded.

### Psychological assessment

The students were subjected to the Persian version of the Depression Anxiety Stress Scale (DASS21). It consists of three self-report scales designed to measure different emotional states. DASS21 questions were adjusted according to their importance and the culture of Iranian students. The DASS21 scale was scored on a four-point scale to assess the extent to which participants experienced each condition over the past few weeks. The scoring method was such that each question was scored from 0 (never) to 3 (very high). Samani (2008) found that the questionnaire has a validity of 0.77 and a Cronbach’s alpha of 0.82 [43].

### Use of social media questionnaire

Among the 13 questions on social media use in the questionnaire, seven were asked on a Likert scale (never, sometimes, often, almost, and always) that examined the problematic use of social media, and six were asked about how much time users spend on social media. Because some items were related to the type of social media platform, which is not available today, and users now use newer social media platforms such as WhatsApp and Instagram, the questionnaires were modified by experts and fundamentally changed, and a 22-item questionnaire was obtained that covered the frequency of using social media. Cronbach’s alpha was equal to 0.705 for the first part, 0.794 for the second part, and 0.830 for all questions [44]. Considering the importance of the problematic use of the social media, six questions about the problematic use were measured separately.

To confirm the validity of the questionnaire, a panel of experts with CVR 0.49 and CVI 0.70 was used. Its reliability was also obtained (0.784) using Cronbach’s alpha coefficient. Finally, the questionnaire was tested in a class with 30 students to check the level of difficulty and comprehension of the questionnaire. Finally, a 22-item questionnaire was obtained, of which six items were about the problematic use of social media and the remaining 16 questions were about the rate and frequency of using social media. Cronbach’s alpha was 0.705 for the first part, including questions about the problematic use of the social media, and 0.794 for the second part, including questions about the rate and frequency of using the social media. The total Cronbach’s alpha for all questions was 0.830. Six questions about the problematic use of social media were measured separately due to the importance of the problematic use of social media. Also, a separate score was considered for each question. The scores of these six questions on the problematic use of the social media were summed, and a single score was obtained for analysis.

### Statistical analysis

Data were analyzed using the Statistical Package for Social Sciences (SPSS) version 26.0 (SPSS Inc., Chicago, IL, USA). The normal distribution of continuous variables was analyzed using the Kolmogorov-Smirnov test, histogram, and P-P diagram, which showed that they are not normally distributed. Descriptive statistics were calculated for all variables. Comparison between groups was done using Mann-Whitney and Kruskal-Wallis non-parametric tests. Multiple linear regression analysis was used to investigate the relationship between mental health, problematic use of social media, and social media use (The result of merging the Frequency of using social media and Time to use social media). Generalized Linear Models (GLM) were used to assess the association between mental health with the use of social media and problematic use of social media. Due to the high correlation (r = 0.585, p = < 0.001) between the use of social media and problematic use of social media, collinearity, we run two separate GLM models. Regression coefficients (β) and adjusted β (β*) with 95% CI and P-value were reported.

## Results

A total of 781 participants completed the questionnaires, of which 64.4% were women and 71.3% were single. The minimum age of the participants was 17 years, the maximum age was 45 years, and about half of them (48.9%) were between 21 and 25 years old. A total of 53.4% of the participants had bachelor’s degrees. The income level of 23.2% of participants was less than five million Tomans (the currency of Iran), and 69.7% of the participants were unemployed. 88.1% were living with their families and 70.8% were studying in non-medical fields. 86% of the participants lived in the city, and 58.9% were in their fourth semester or higher. Considering that the research was conducted in a Lorish Province, 43.8% of participants were from the Lorish ethnicity.

The mean total score of mental health was 12.30 with a standard deviation of 30.38, and the mean total score of social media was 14.5557 with a standard deviation of 7.74140.

Table 1 presents a comparison of the mean problematic use of social media and mental health with demographic variables. Considering the non-normality of the hypothesis H0, to compare the means of the independent variables, Mann-Whitney non-parametric tests (for the variables of gender, the field of study, academic semester, employment status, province of residence, and whether it is rural or urban) and Kruskal Wallis (for the variables age, ethnicity, level of education, household income and marital status). According to the obtained results, it was found that the score of problematic use of social media is significantly higher in women, the age group less than 20 years, unemployed, non-native students, dormitory students, and students living with friends or alone, Fars students, students with a household income level of fewer than 7 million Tomans(Iranian currency), and single, divorced, and widowed students were higher than the other groups(P < 0.05).

**Table 1:** Comparing the average problematic use of social media and mental health with demographic variables (n = 781)

				Problematic Social Media		Mental Health	
Variable		Frequency	Percent	M ± SD	P	M ± SD	P
Gender	Female	503	64.4	15.03(4.84)	0.**011**	31.88(28.62)	0.**016**
	Male	278	35.6	14.17(4.77)		26.93(27.31)	
Age	≤ 20	168	21.5	16.40(4.83)	0.**000**	40.03(29.19)	0.**000**
	21–25	382	48.9	14.11(4.70)		25.20(25.66)	
	≥ 26	231	29.6	14.52(4.77)		31.03(29.72)	
Major	Medical	228	29.2	14.50(4.55)	0.517	23.85(23.59)	0.**000**
	Non-Medical	553	70.8	14.82(4.93)		32.70(29.59)	
semester	1–3	317	40.6	14.93(5.03)	0.404	31.07(28.71)	0.394
	≥ 4	460	58.9	14.58(4.67)		29.55(28.04)	
Employ status	Employed	233	29.8	14.07(4.77)	0.**008**	26.42(27.51)	0.**003**
	Unemployed	544	69.7	15.01(4.84)		31.57(28.33)	
Province of residence	Lorestan	308	39.4	14.00(4.78)	0.**000**	27.68(29.84)	0.**001**
	Other	469	60.1	15.22(4.76)		31.79(27.01)	
Residence status	Urban	671	85.9	14.67(4.78)	0.559	29.72(27.72)	0.528
	rural	110	14.1	15.08(5.11)		32.50(31.29)	
Residential status	With Family	688	88.1	14.49(4.78)	0.**000**	28.95(27.69)	0.**000**
	In dormitory	61	7.8	15.85(4.77)		48.12(36.02)	
	other	32	4.1	17.53(5.00)		33.80(26.84)	
Race	Lor	342	43.8	13.94(4.69)	0.**000**	27.67(28.79)	0.**000**
	Fars	273	35.0	15.25(4.94)		32.32(28.68)	
	Other	166	21.3	15.46(4.70)		31.53(26.06)	
Education	Associate’s degree	141	18.1	14.68(4.82)	0.814	31.07(28.26)	0.814
	Bachelor’s degree	417	53.4	14.62(4.80)		29.98(28.34)	
	Master’s Degree	139	17.8	14.96(4.80)		28.04(26.59)	
	Doctorate	84	10.8	14.91(5.09)		32.64(30.55)	
Household income (Toman currency)	≤ 5 Million	181	23.2	15.70(4.88)	0.**000**	36.99(27.97)	0.**000**
	5–7 Million	170	21.8	16.15(4.79)		36.14(28.80)	
	7–10 Million	240	30.7	13.65(4.48)		22.11(25.47)	
	≥ 10 Million	190	24.3	13.86(4.76)		28.29(28.68)	
Marital status	Single	557	71.3	15.01(4.66)	0.**000**	31.07(27.27)	0.**000**
	Married	207	26.5	13.53(4.81)		24.10(27.35)	
	other	17	2.2	19.76(6.03)		2.23(32.20)	

**Statistics:** Kruskal Wallis tests and #Mann–Whitney U test. Bold text indicates statistically significant results at an alpha of 5%

By comparing the mean score of mental health with demographic variables using non-parametric Mann-Whitney and Kruskal Wallis tests, it was found that there is a significant difference between the variable of poor mental health and all demographic variables (except for the semester variable), residence status (rural or urban) and education level. (There was a significant relationship (P < 0.05). In such a way that the mental health condition was worse in women, age group less than 20 years old, non-medical science, unemployed, non-native, and dormitory students. Also, Fars students, divorced, widowed, and students with a household income of fewer than 5 million Tomans (Iranian currency) showed poorer mental health status. (Table 1).

The final model shows that marital status, field, and household income were significantly associated with lower DASS21 scores (a lower DASS21 score means better mental health status). Being single (β* = -23.03, 95% CI: (-33.10, -12.96), being married (β* = -38.78, 95% CI: -51.23, -26.33), was in Medical sciences fields (β* = -8.15, 95% CI: -11.37, -4.94), and have income 7–10 million (β* = -5.66, 95% CI: -9.62, -1.71) were significantly associated with lower DASS21 scores (a lower DASS21 score means better mental health status). Problematic use of social media (β* = 3.54, 95% CI: (3.23, 3.85) was significantly associated with higher mental health scores (a higher DASS21 score means worse mental health status). (Table 2)

**Table 2:** The univariable and multivariable generalized linear models (GLM) to determine the association between problematic use of social media and mental health (n = 781)

Variables	Univariable		Multivariable	
	β (95% CI)	P-value	β* (95% CI)	P-value
**Age (year)**				
< 20 year	9.00 (3.51, 14.49)	0.001*	-	-
21–25 year	-5.82 (-10.33, -1.30)	0.012*	-	-
≥ 26 year	Reference	-	-	-
**Gender**				
Female	4.94 (0.82, 9.06)	0.019*	-	-
Male	Reference	-	-	-
**Marital status**				
Single	-41.16 (-54.36, -27.96)	< 0.001*	-23.03 (-33.10, -12.96)	< 0.001**
Married	-48.12 (-61.65, -34.60)	< 0.001*	-26.12 (-36.49, -15.75)	< 0.001**
Other	Reference	-		
**Education**				
Associate’s degree	-1.56 (-4.86, -2.83)	0.687	-	-
Bachelor’s degree	-2.66 (-9.27, 3.94)	0.430	-	-
Master’s Degree	-4.60 (-12.23, 3.03)	0.238		
Doctorate	Reference	-		
**Field**				
Medical sciences	-8.85 (-13.16, -4.54)	< 0.001*	-8.15 (-11.37, -4.94)	< 0.001**
Other	Reference	-	Reference	-
**Ethnicity**				
Lor	-3.85 (-9.07, 1.36)	0.148	-	-
Fars	0.79 (-4.63, 6.22)	0.775	-	-
Other	Reference	-	-	-
**Semester**				
1–3	1.51 (-2.53, 5.56)	0.463	-	-
≥ 4	Reference	-	-	-
**Employment status**				
Employed	-5.14 (-9.45, -0.84)	0.019*	-	-
Unemployed	Reference	-	-	-
**Household income**				
≤ 5 Million	8.70 (3.09, 14.30)	0.002*	1.77 (-2.49, 6.04)	0.415
5–7 Million	7.84 (2.15, 13.54)	0.007*	-1.36 (-5.72, 2.98)	0.539
7–10 Million	-6.17 (-11.41, -0.93)	0.021*	-5.66 (-9.62, -1.71)	0.005**
≥ 10 Million	Reference	-	Reference	-
**Province**				
Lorestan	-4.10 (-8.14, -0.05)	0.047*	-	-
Other	Reference	-	-	-
**Residence**				
Urban	-2.78 (-8.46, 2.90)	0.338	-	-
Rural	Reference	-	-	-
**Housing**				
With Family	-4.84 (-12.16, 2.47)	0.194	-	-
In dormitory	14.32 (2.36, 26.28)	0.019*	-	-
other	Reference	-	-	-
Problematic use of social media	3.79 (3.48, 4.10)	< 0.001*	3.54 (3.23, 3.85)	< 0.001**

β*: Adjusted β

*Variables with P-value < 0.2 which were entered in multiple GLM

** Variables with P-value < 0.05 in multiple GLM

**Statistics:** Univariable and multivariable generalized linear models (GLM)

Age, income, and use of social media (β* = 1.02, 95% CI: 0.78, 1.25) were significantly associated with higher DASS21 scores (a higher DASS21 score means worse mental health status). Marital status and field were significantly associated with lower DASS21 scores (a lower DASS21 score means better mental health status). Age groups < 20 years (β* = 6.36, 95% CI: 0.78, 11.95) and income group < 5 million (β* = 6.58, 95% CI: 1.47, 11.70) increased mental health scores. Being single (β* = -34.72, 95% CI: -47.06, -38.78), being married (β* = -38.78, 95% CI: -51.23, -26.33) and in medical sciences fields (β* = -8.17, 95% CI: -12.09, -4.24) decreased DASS21 scores. (Table 3)

**Table 3:** The univariable and multivariable generalized linear models (GLM) to determine the association between use of social media and mental health (n = 781)

Variables	Univariable		Multivariable	
	β (95% CI)	P-value	β* (95% CI)	P-value
**Age (year)**				
< 20 year	9.00 (3.51, 14.49)	0.001*	6.36 (0.78, 11.95)	0.025**
21–25 year	-5.82 (-10.33, -1.30)	0.012*	-3.60 (-8.04, 0.83)	0.111
≥ 26 year	Reference	-	Reference	-
**Gender**				
Female	4.94 (0.82, 9.06)	0.019*	-	-
Male	Reference	-	-	-
**Marital status**				
Single	-41.16 (-54.36, -27.96)	< 0.001*	-34.72 (-47.06, -38.78)	< 0.001**
Married	-48.12 (-61.65, -34.60)	< 0.001*	-38.78 (-51.23, -26.33)	< 0.001**
Other	Reference	-	Reference	-
**Education**				
Associate’s degree	-1.56 (-4.86, -2.83)	0.687	-	-
Bachelor’s degree	-2.66 (-9.27, 3.94)	0.430	-	-
Master’s Degree	-4.60 (-12.23, 3.03)	0.238		
Doctorate	Reference	-		
**Field**				
Medical sciences	-8.85 (-13.16, -4.54)	< 0.001*	-8.17 (-12.09, -4.24)	< 0.001**
Other	Reference	-	Reference	
**Ethnicity**				
Lor	-3.85 (-9.07, 1.36)	0.148	-	-
Fars	0.79 (-4.63, 6.22)	0.775	-	-
Other	Reference	-	-	-
**Semester**				
1–3	1.51 (-2.53, 5.56)	0.463	-	-
≥ 4	Reference	-	-	-
**Employment status**				
Employed	-5.14 (-9.45, -0.84)	0.019*	-	-
Unemployed	Reference	-	-	-
**Household income**				
≤ 5 Million	8.70 (3.09, 14.30)	0.002*	6.58 (1.47, 11.70)	0.012**
5–7 Million	7.84 (2.15, 13.54)	0.007*	3.48 (-1.73, 8.69)	0.190
7–10 Million	-6.17 (-11.41, -0.93)	0.021*	-4.64 (-9.45, 0.17)	0.059
≥ 10 Million	Reference	-	-	-
**Province**				
Lorestan	-4.10 (-8.14, -0.05)	0.047*	-	-
Other	Reference	-	-	-
**Residence**				
Urban	-2.78 (-8.46, 2.90)	0.338	-	-
Rural	Reference	-	-	-
**Housing**				
With Family	-4.84 (-12.16, 2.47)	0.194	-	-
In dormitory	14.32 (2.36, 26.28)	0.019*	-	-
other	Reference	-	-	-
Use of social media	1.30 (1.06, 1.54)	< 0.001*	1.02 (0.78, 1.25)	< 0.001**

β*: Adjusted β

*Variables with P-value < 0.2 which were entered in multiple GLM

** Variables with P-value < 0.05 in multiple GLM

**Statistics:** Univariable and multivariable generalized linear models (GLM)

## Discussion

The main purpose of this study was to determine the relationship between social media use and mental health among students during the COVID-19 pandemic.

University students are more reliant on social media because of the limitations of in-person interactions [45]. Since social media has become more and more vital to the social lives of university students during the pandemic, students may be at increased risk of social media addiction, which may be harmful to their mental health [14].

During non-adulthood, peer relations and approval are critical and social media seems to meet these needs. For example, connection and communication with friends make them feel better and happier, especially during the COVID-19 pandemic and national lockdowns where face-to-face communication was restricted [46]. Kele’s study showed that the COVID-19 pandemic has increased the time spent on social media, and the frequency of online activities [47].

Because of the COVID-19 pandemic, e-learning became the only sustainable option for students [13]. This abrupt transition can lead to depression, stress, or anxiety for some students due to insufficient time to adjust to the new learning environment. The role of social media is also important to some university students [48].

Staying at home, having nothing else to do, and being unable to go out and meet with friends due to the lockdown measures increased the time spent on social media and the frequency of online activities, which influenced their mental health negatively [49]. These reasons may explain the findings of previous studies that found an increase in depression and anxiety among adolescents who were healthy before the COVID-19 pandemic [50].

According to the results, there was a statistically significant relationship between social media use and mental health in students, in such a way that one Unit increase in the score of social media use enhanced the score of mental health. These two variables were directly correlated. Consistent with the current study, many studies have shown a significant relationship between higher use of social media and lower mental health in students [45, 51–54].

Inconsistent with the findings of the present study, some previous studies reported the positive effect of social media use on mental health [55–57]. The differences in findings could be attributed to the time and location of the studies. Anderson’s study in France in 2018 found no significant relationship between social media use and mental health. This may be because of the differences between the tools for measuring the ability to detect fake news and health literacy and the scales of the research [4].

The present study showed that the impact of using social media on the mental health of students was higher than Lebni’s study, which was conducted in 2020 [25]. Also, in Dost Mohammad’s study in 2018, the effect of using social media on the mental health of students was reported to be lower than in the present study [58]. Entezari’s study in 2021, was also lower than the present study [59]. It seems that the excessive use of social media during the COVID-19 pandemic was the reason for the greater effects of social media on students’ mental health.

The use of social media has positive and negative characteristics. Social media is most useful for rapidly disseminating timely information via widely accessible platforms [4]. Among the types of studies, at least one shows an inverse relationship between the use of social media and mental health [53]. While social media can serve as a tool for fostering connection during periods of physical isolation, the mental health implications of social media being used as a news source are tenuous [45].

The results of the GLM analysis indicated that there was a statistically significant relationship between the problematic use of social media and mental health in students in such a way that one-unit increase in the score of problematic use of social media enhanced the mental health score, and it was found that the two variables had a direct relationship. Consistent with our study, Boer’s study showed that problematic use of social media may highlight the potential risk to adolescent mental health [60]. Malaeb also reported that the problematic use of social media had a positive relationship with mental health [61], but that study was conducted on adults and had a smaller sample size before the COVID-19 pandemic.

Saputri’s study found that excessive social media use likely harms the mental health of university students since students with higher social media addiction scores had a greater risk of experiencing mild depression [62]. A systematic literature review before the COVID-19 pandemic (2019) found that the time spent by adolescents on social media was associated with depression, anxiety, and psychological distress [63]. Marino’s study (2018) reported a significant correlation between the problematic use of social media by students and psychological distress [64].

Social media has become more vital for students’ social lives owing to online education during the COVID-19 pandemic. Therefore, this group is more at risk of addiction to social media and may experience more mental health problems than other groups. Lebni also indicated that students’ higher use of the Internet led to anxiety, depression, and adverse mental health, but the main purpose of the study was to investigate the effects of such factors on student’s academic performance [25]. Previous studies indicated that individuals who spent more time on social media had lower self-esteem and higher levels of anxiety and depression [65, 66]. In the present study, students with higher social media addiction scores were at higher mental health risk. Such a finding was consistent with research by Gao et al., who found that the excessive use of social media during the pandemic had adverse effects on social health [14]. Cheng et al. indicated that using the Internet, especially for communication with people, can harm mental health by changing the quality of social relationships, face-to-face communication, and changes in social support [24].

A reason for the significant relationship between social media use and mental health in students during the COVID-19 pandemic in the present study was probably the students’ intentional or unintentional use of online communication. Unfortunately, social media published information, which might be incorrect, in this pandemic that caused public fear and threatened mental health.

During the pandemic, social media played essential roles in learning and leisure activities. Due to electronic education, staying at home, and long leisure time, students had more time, frequency, and opportunities to use social media in this pandemic. Such a high reliance on social media may threaten student’s mental health. Lee et al. conducted a study during the COVID-19 pandemic and confirmed that young people who used social media had higher symptoms of depression and loneliness than before the COVID-19 pandemic [67].

The present study showed that there was a significant positive relationship between problematic use of social media and gender, so that women were more willing to use social media, probably because they had more opportunities to use social media as they stayed at home more than men; hence, they were more exposed to problematic use of social media. Consistent with our study, Andreassen reported that being a woman was an important factor in social media addiction [68]. In contrast to our study, Azizi’s study in Iran showed that male students use social media significantly more than female students, possibly due to differences in demographic variables in each population [69].

Moreover, there was a significant relationship between age and problematic use of social media in that people younger than 20 were more willing to use social media in a problematic way. Consistent with the present study, Perrin also indicated that younger people further used social media [70].

According to the findings, unemployed students used social media more than employed ones, probably because they had more time to spend in virtual space, leading to higher use and the possibility of problematic use of social media [71].

Moreover, non-native students were more willing to use the social media probably because students who lived far away from their families used social media problematically due to the lack of family control over hours of use and higher opportunities [72] .

The results showed that rural students have a greater tendency to use social Medias than urban students. Inconsistent with this finding, Perrin reported that urban people were more willing to use the social media. The difference was probably due to different research times and places or different target groups [70].

According to the current study, people with low household income were more likely to use social media, most likely because low-income people seek free information and services due to a lack of access to facilities and equipment in the real world or because they seek assimilation with people around them. Inconsistent with our findings, Hruska et al. reported that people with high household income levels made much use of social media [73], probably because of cultural, economic, and social differences or different information measurement tools.

Furthermore, single, divorced, and widowed students used social media more than married students. This is because they spend more time on social media due to the need for more emotional attention, the search for a life partner, or a feeling of loneliness. This also led to the problematic use of social media [74].

According to the results, Fars people used social media more than other ethnic groups, but this difference was insignificant. This finding was consistent with Perrin’s study, but the population consisted of people aged 18 to 65 [70].

In the current study, there was a significant relationship between gender and mental health, so that women had lower mental health than men. The difference was in health sociology. Consistent with the present study, Ghasemi et al. indicated that it appeared necessary to pay more attention to women’s health and create an opportunity for them to use health services [75].

The findings revealed that unemployed students had lower mental health than employed students, most likely because unemployed individuals have lower mental health due to not having a job and being economically dependent on others, as well as feeling incompetent at times. Consistent with the present study, Bialowolski reported that unemployment and low income caused mental disorders and threatened mental health [76].

According to this study, non-native students have lower mental health than native students because they live far from their families. The family plays an imperative role in improving the mental health of their children, and mental health requires their support. Also, the economic, social, and support problems caused by being away from the family have endangered their mental health [77].

Another important factor of the current study was that married people had higher mental health than single people. In addition, divorced and widowed students had lower mental health [78]. Possibly due to the social pressure they suffer in Iranian society. Furthermore, they received lower emotional support than married people. Therefore, their lower mental health seemed logical [79–81]. A large study in a European population also reported differences in the likelihood of mood, anxiety, and personality disorders between separated/divorced and married mothers [82].

A key point confirmed in other studies is the relationship between low incomes with mental health. A meta-analysis by Lorant indicated that economic and social inequalities caused mental disorders [83]. Safran also reported that the probability of developing mental disorders in people with low socioeconomic status is up to three times higher than that of people with the highest socioeconomic status [84]. Bialowolski’s study was consistent with the current study but Bialowolski’s study examined employees [76].

## Limitation

The present study was conducted during the COVID-19 pandemic and therefore had limitations in accessing students. Another limitation was the use of self-reporting tools. Participants may show positive self-presentation by over- or under-reporting their social media-related behaviors and some mental health-related items, which may directly or indirectly lead to social desirability bias, information bias, and reporting bias. Small sample sizes and convenience sampling limit student population representativeness and generalizability. This study was based on cross-sectional data. Therefore, the estimation results should be seen as associative rather than causative. Future studies would need to investigate causal effects using a longitudinal or cohort design, or another causal effect research design.

## Conclusion

The findings of this study indicated that the high use of social media affected students’ mental health. Furthermore, the problematic use of the social media had a direct relationship with mental health. Variables such as age, gender, income level, marital status, and unemployment of non-native students had significant relationships with social media use and mental health. Despite the large amount of evidence suggesting that social media harms mental health, more research is still necessary to determine the cause and how social media can be used without harmful effects. It is imperative to better understand the relationship between social media use and mental health symptoms among young people to prevent such a negative outcome.

## Data Availability

The datasets used and/or analyzed during the current study are available from the corresponding author on reasonable request.
